# Mesenchymal stromal/stem cells and their extracellular vesicles in liver diseases: insights on their immunomodulatory roles and clinical applications

**DOI:** 10.1186/s13578-023-01122-3

**Published:** 2023-09-05

**Authors:** Qian Huai, Cheng Zhu, Xu Zhang, Hanren Dai, Xiaolei Li, Hua Wang

**Affiliations:** 1 Department of Oncology, The First Affiliated Hospital of Anhui Medical University, Hefei, 230022 China; 2 Inflammation and Immune Mediated Diseases Laboratory of Anhui Province, Anhui Medical University, Hefei, 230032 China

**Keywords:** Mesenchymal stromal cell, Extracellular vesicles, Liver disease, Immunomodulation, Clinical application

## Abstract

Liver disease is a leading cause of mortality and morbidity that is rising globally. Liver dysfunctions are classified into acute and chronic diseases. Various insults, including viral infections, alcohol or drug abuse, and metabolic overload, may cause chronic inflammation and fibrosis, leading to irreversible liver dysfunction. Up to now, liver transplantation could be the last resort for patients with end-stage liver disease. However, liver transplantation still faces unavoidable difficulties. Mesenchymal stromal/stem cells (MSCs) with their broad ranging anti-inflammatory and immunomodulatory properties can be effectively used for treating liver diseases but without the limitation that are associated with liver transplantation. In this review, we summarize and discuss recent advances in the characteristics of MSCs and the potential action mechanisms of MSCs-based cell therapies for liver diseases. We also draw attention to strategies to potentiate the therapeutic properties of MSCs through pre-treatments or gene modifications. Finally, we discuss progress toward clinical application of MSCs or their extracellular vesicles in liver diseases.

## Introduction

Liver diseases have become a significant health issue worldwide and urgently require the development of novel therapeutic methods [1]. Liver dysfunctions are classified into acute and chronic diseases, which comprise a heterogeneous group of pathological features and a high mortality rate [2]. Various factors, including viral and bacterial infections, autoimmune responses, diabetes, drugs, alcohol abuse, and fat deposition, may cause chronic inflammation and fibrosis, leading to irreversible liver dysfunction. Chronic liver diseases slowly develop into end-stage liver diseases, including decompensated cirrhosis and liver failure, if not controlled properly and promptly. Currently, the most effective treatment for end-stage liver diseases is liver transplantation. Liver transplantation remains the only available approach to improve survival but is restricted by a shortage of organ resources, rejection after transplantation, and heavy financial costs. To address the shortage of donor liver organs for orthotopic liver transplantation, cell therapy in liver disease has emerged as a promising regenerative treatment.

Mesenchymal stromal/stem cells (MSCs)-based cell therapies have gradually become a hot topic for promoting liver regeneration and repairing liver injury in various liver diseases. MSCs are a type of multipotent stromal cells with self-renewal capacity that can be found in various tissue of the body, such as bone marrow, adipose tissue, and umbilical cord. They possess the ability to differentiate into multiple cell types, including osteoblasts, chondrocytes and adipocytes [3]. MSCs also exhibits immunomodulatory, anti-fibrotic, and liver regenerative properties, making them attractive for potential therapeutic applications in regenerative medicine and various liver disorders. These cells have been extensively studied owing to their regenerative potential and their role in liver repair and immunoregulation. This review summarizes the main therapeutic uses of MSCs in liver diseases, highlights their mechanisms of action in immunoregulation, and discusses the extrinsic inflammatory cues that elicit the immunoregulatory properties in these cells. We also provide some perspectives on possible strategies that can augment the therapeutic effects of MSCs and their extracellular vesicles (EVs) in liver diseases.

## Characteristics of MSCs

**Fig. 1:**
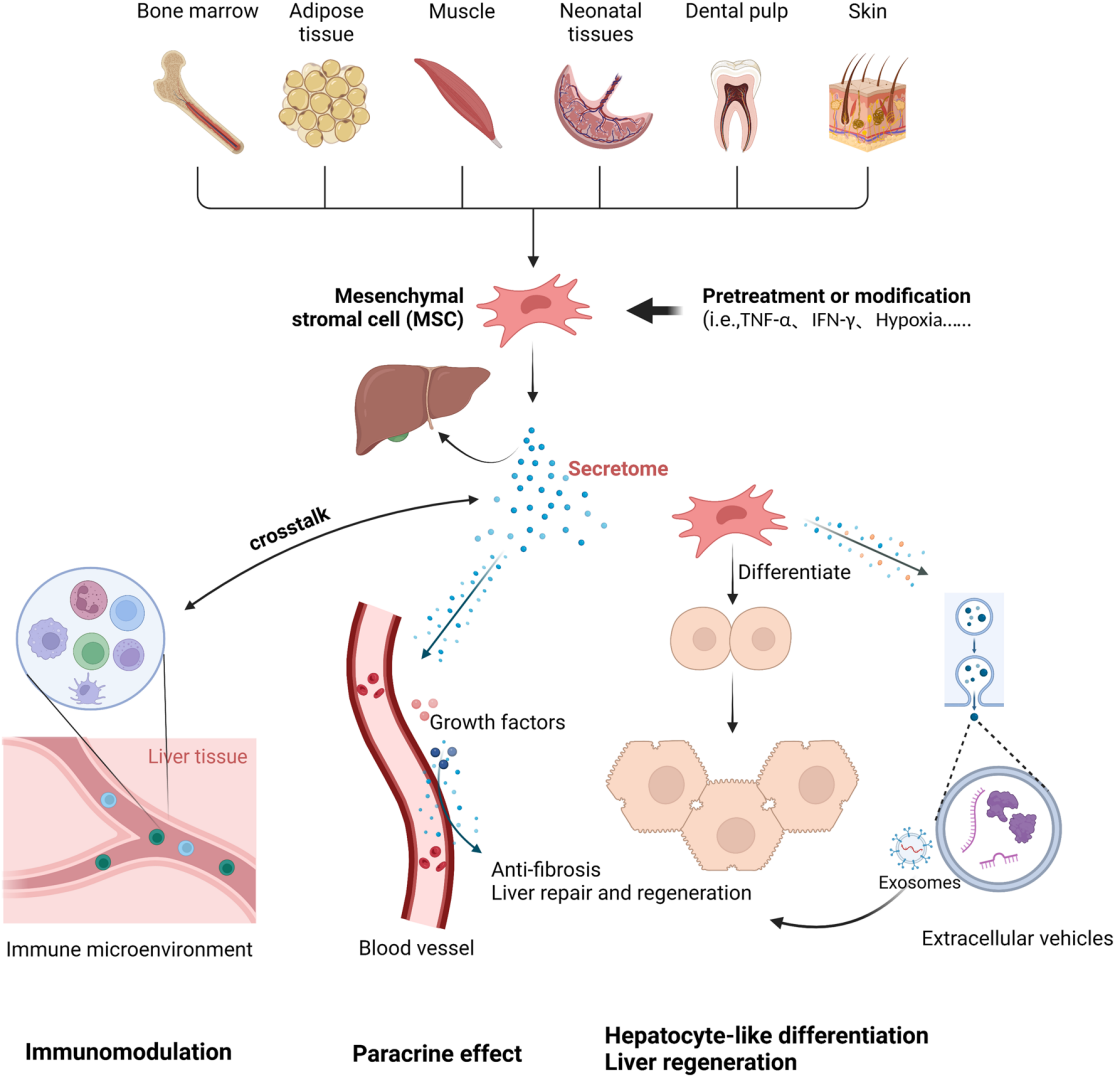
Potential mechanisms of action of MSCs in liver diseases. MSCs from different sources exhibit potential therapeutic effects in liver diseases through various mechanisms such as immunomodulation, paracrine factors, hepatocyte-like differentiation and regeneration, anti-fibrosis and the utilization of MSCs-derived exosomes

MSCs were initially identified from cells derived from bone marrow stroma in the late 1960s on account of self-renewal and colony formation capabilities [4], and then these cells were systematically characterized by fibroblast-like cells with the capacity of attachment-dependent growth, surface expression of various markers and tri-lineage differentiation potential into osteoblasts, chondrocytes and adipocytes. MSCs are pluripotent stem cells with self-renewal capacity and multi-directional differentiation. Although they are originally identified and isolated from bone marrow cells, MSCs have also been successfully isolated and expanded from many different tissues, including adipose tissue, amniotic fluid, skin, tooth pulp, muscle, placenta and umbilical cord blood, as well as umbilical cord [3] (Fig. 1). Notably, MSCs from different sources display similar expression profiles for MSCs surface markers and similar morphological features in culture. The phenotypic profiles of both human and mouse MSCs are generally positive for CD29, CD73, CD90 and CD105, and negative for CD31, CD45 or markers of the hematopoietic lineage [5]. Nevertheless, several studies have demonstrated that MSCs from different sources differ in immunomodulatory properties and biological characteristics [6–8]. In addition, umbilical cord-derived MSCs (UC-MSCs) have been considered as a particularly promising population thanks to their capacity for higher proliferation and stronger immunomodulation than MSC populations obtained from other sources [9]. Thus, ex vivo-expanded human UC-MSCs have emerged as an important therapeutic candidate for the treatment of many human inflammatory diseases and tissue damages in the last few years.

## Potential mechanisms of MSCs-based therapy in liver diseases

### Hepatocyte-like differentiation and liver regeneration

MSCs have the potential for self-renewal and differentiation into multiple cell lineages, including hepatocytes. Extensive studies have demonstrated that MSCs may serve as a novel source for the propagation of hepatocyte-like cells suitable for cell therapy in liver diseases. MSCs derived from bone marrow have the potential to differentiate into hepatocytes, which could be induced into hepatocyte-like cells with liver-specific morphology and function under appropriate conditions to promote liver tissue regeneration [10]. It was demonstrated that both MSCs-derived hepatocyte-like cells and MSCs can engraft recipient liver, differentiate into functional hepatocytes, and rescue liver failure via intrasplenic or intravenous routes. Noticeability, compared to MSCs-derived hepatocyte-like cells, MSCs could exhibit greater resistance to reactive oxygen species (ROS), reduce oxidative stress in recipient mice, and accelerate repopulation of hepatocytes after liver damage.

*N*-Acetylcysteine (NAC) has been the only clinically approved antidote recommended and is the most beneficial therapy for acetaminophen (APAP) overdose patients at risk of liver damage. However, NAC has a limited therapeutic window and prolonged NAC treatment delays liver regeneration by inducing hepatocyte vacuolation. Both human and mouse MSCs have been shown to significantly improve the mouse survival rate for APAP-induced fulminant liver failure, suggesting that the administration of MSCs may be another therapeutic option for APAP poisoning. Additionally, MSCs have similar effects on reduced hepatocyte necrosis and granulocytic myeloid-derived suppressor cells (MDSCs) infiltration but enhanced the proportion of regenerative monocytic MDSCs when compared to NAC. Mechanistically, MSCs attenuate hepatocyte necrosis by secreting hepatocyte growth factor (HGF) [11]. In rat models of post-hepatectomy liver failure, MSCs transplantation was shown to enhance liver regenerative capacities by facilitating glucose and lipid metabolism [12]. Conditioned medium (CM) produced by human amnion-derived MSCs had the potential to increase hepatic stem/progenitor cell differentiation, demonstrating that soluble factors secreted by those cells are potentially responsible for the reaction [13]. In addition, using large animal (pig) model of repeated biliary obstruction followed by partial hepatectomy, MSCs transplantation can promote growth of liver tissue without any effect on liver function [14]. Several groups have also reported that placenta-derived mesenchymal stem cells transplantation can regenerate the liver in different liver injury model through antifibrotic, antioxidant and autophagic mechanisms [15, 16].

Due to the multi-differentiation potential of MSCs, targeting the hepatic differentiation potential of MSCs is expected to become an alternative strategy for treating liver diseases. Recently, Choi et al. [17] has offered an efficient and xeno-free conditioned hepatic differentiation protocol for differentiating amnion-derived MSCs into hepatic progenitor cells (HPCs), called AM-HPC cells. When these AM-HPC cells were transplanted into the liver failure mouse model, they settled in the damaged livers and differentiated into hepatocytes, suggesting that AM-HPCs are promising cells for treating liver disease. Additionally, a biomimetic microenvironment constructed by hE-cadherin-coated poly (lactic-co-glycolic) acid (PLGA) in engineered multicellular aggregates facilitated endoderm differentiation and subsequent hepatic differentiation of human MSCs [18]. Hepatic differentiation of human MSCs was induced by a biomimetic microenvironment consisting of these engineered aggregates and a cocktail of specific cytokines, suggesting that cell aggregates could enhance the efficiency of MSCs expansion and differentiation. These findings support the notion that mechanical forces and the microenvironment’s architecture influence the efficiency of MSCs differentiation into hepatocytes.

### Immunomodulatory effects

**Fig. 2:**
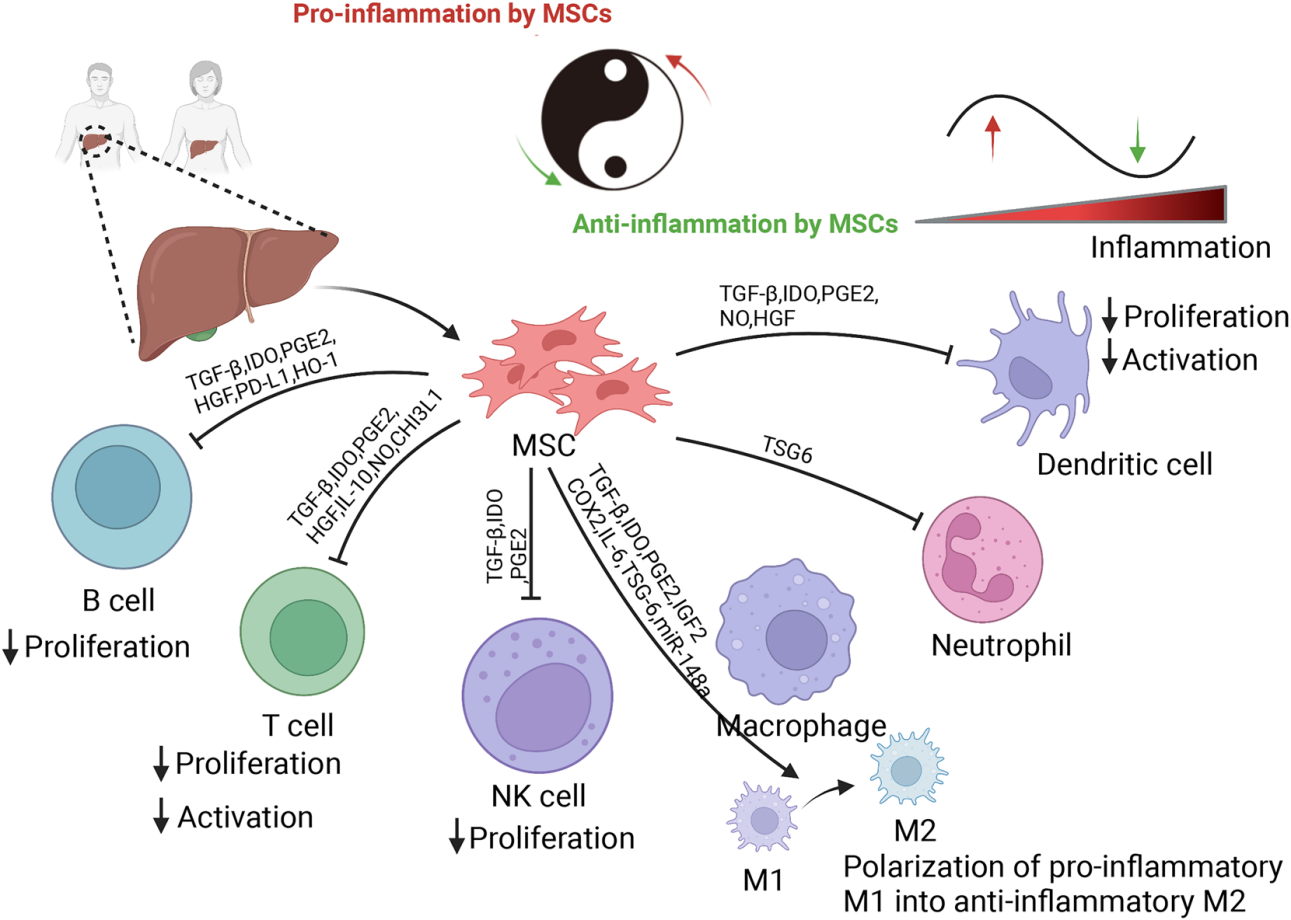
Immunomodulatory properties of MSCs. At inflamed and injured tissue sites, MSCs can regulate the immune homeostasis and their immunomodulatory functions are predominantly exerted through directly or indirectly with innate and adaptive immune cells such as macrophages, neutrophils, natural killer (NK) cells, dendritic cells (DCs), T lymphocytes, and B lymphocytes, and thus suppressing the immune responses and inflammation to promote tissue homeostasis. In fact, different inflammatory environment can lead to markedly different response to MSC treatment, thus the ultimate immunomodulatory impact may be determined by the balance between anti-inflammatory and pro-inflammatory cytokines in the milieu in which MSCs present

MSCs-based therapies have been widely utilized for the treatment of diverse acute and chronic liver diseases, due to the potent immunomodulatory functions of MSCs [19]. At inflamed and injured tissue sites, MSCs can regulate the immune homeostasis and their immunomodulatory functions are predominantly exerted through directly or indirectly with innate and adaptive immune cells such as macrophages, neutrophils, natural killer (NK) cells, dendritic cells (DCs), T lymphocytes, and B lymphocytes, and thus suppressing the immune responses and inflammation to promote tissue homeostasis [20, 21] (Fig. 2).

### Immunomodulatory effect of MSCs on innate immunity

Inflammation is one of the most characteristic features of different liver diseases and plays key roles in the pathogeneses of liver diseases [22]. MSCs modulate innate and adaptive immune responses. Monocytes arriving at an inflammatory environment can be classified into activated M1 (pro-inflammatory subtype) macrophages or alternatively activated M2 (anti-inflammatory subtype) macrophages depending on microenvironmental cues. MSCs have been reported to trigger polarization of M1 macrophages toward M2 macrophages both in vivo and ex vivo [23–25]. This polarizing effect of MSCs on M2 macrophages is driven by the ability of MSCs to secrete soluble factors such as indoleamine-2,3-dioxygenase (IDO), insulin-like growth factor-2, and prostaglandin E2 (PGE2) which have shown to play an important role in the switch between proinflammatory and anti-inflammatory macrophages [26–28]. Moreover, different sources of MSCs could regulate macrophages mainly through EVs. A study by Liu et al. [29] showed that administration of MSC-derived exosomes in the model of acute liver failure (ALF) induced by lipopolysaccharide and D-galactosamine (LPS/GalN) significantly ameliorated ALF. Other studies have showed that exosomes-shuttled miR-17 can suppress NOD-like receptor thermal protein domain associated protein 3 (NLRP3) inflammasome activation by the targeting thioredoxin-interacting protein in hepatic macrophages. Congruously, another group has also reported [30] that MSCs-derived exosomes can modulate macrophage phenotype to regulate inflammatory microenvironment in liver and repair the injury. Mechanistically, MSC-derived exosomes protect liver fibrosis via delivering miR-148a to target Kruppel-like factor 6/signal transducer and activator of transcription 3 (STAT3) pathway in macrophages. In mouse model of CCl_4_-induced acute liver injury (ALI), Zhou et al. [31] demonstrated that the characteristics of heterogeneous monocyte-derived macrophages (MoMF) during different periods of ALI and revealed their functional changes after MSCs treatment. Single-cell RNA sequencing data revealed distinct macrophage populations and indicated MSCs could effectively alleviate ALI via restoring liver macrophages homeostasis and attenuating the recruitment of neutrophils by reducing the expression of C-X-C motif chemokine ligand 2 of MoMF.

Neutrophils are the most abundant circulating leukocytes in the human body and the first responder against invading pathogens or other danger signals. As one of the effectors in the innate immune response, they have several immune functions, including phagocytosis, ROS production and degranulation, as well as the formation and release of neutrophil extracellular traps (NETs) [32]. When liver tissue damage occurs, neutrophils are recruited from the peripheral blood to respond to chemokines released from the injured liver, and the infiltration of neutrophils is commonly observed, especially in mice and patients with alcoholic steatohepatitis [33, 34]. Several studies have reported that MSCs isolated from bone marrow alleviate alcohol-induced liver injury via suppressing hepatic neutrophil and macrophage infiltration, and oxidative stress [35–37]. However, the underlying mechanism by which MSCs alleviate alcohol-induced liver injury by inhibiting hepatic neutrophil and macrophage infiltration remains unclear. Another study demonstrated that MSCs can alleviate alcohol-induced liver injury in mice via release of tumor necrosis factor (TNF) stimulating gene-6 (TSG6) and suppression of STAT3 activation, whereas MSCs injected into mice with alcoholic hepatitis do not engraft the liver [38]. Notably, MSCs can also inhibit neutrophils infiltration into sites of inflammation in a TSG-6-dependent manner [39]. For example, Ding et al. [40] demonstrated that UC-MSCs primed by interferon-γ (IFN-γ) and TNF-α (MSCs-IT) represent remarkable therapeutic efficacy on imiquimod-induced psoriasis-like inflammation in mice. Importantly, MSCs-IT alleviated murine psoriasis-like inflammation via producing TSG-6, which inhibited neutrophil infiltration. Nevertheless, the interrelationship between neutrophils and TSG-6 in MSCs in alcohol-induced liver injury still needs to be studied.

### Immunomodulatory effect of MSCs on adaptive immunity

DCs, are the most effective type of antigen-presenting cells with the ability to acquire, process, and present antigens, which are fundamental for an effective adaptive immune response. Hepatic DCs represent a unique and multifaceted subset of antigen-presenting leukocytes that orchestrate specified immune responses in the liver. The maturation and activation of DCs can be limited by MSCs, leading to the suppression of T cell activation. MSCs can also induce tolerogenic and regulatory DCs to inhibit T cell proliferation and to induce regulatory T cells (Tregs) with the help of interleukin (IL)-6, transforming growth factor-β (TGF-β), HGF, PGE2, and IDO [41, 42]. Notably, NK cells not only perform critical roles in host defense against pathogens and tumors through their natural cytotoxicity and cytokine production, but also act as regulatory cells by engaging in reciprocal interactions with other types of liver cells through cell-to-cell contact and the production of cytokines [43]. MSCs can not only effectively inhibit IL-2-induced NK cell proliferation but also prevent cytotoxic activity and cytokine production with the help of IDO and PGE2, significantly reducing the levels of pro-inflammatory cytokines and reducing the infiltration of inflammatory cells in the liver.

T and B lymphocytes are the main participants of adaptive immune responses. MSCs can suppress the activation and proliferation of T cells either by secreting soluble factors or by directly interacting with T cells. Several studies have reported that a panel of cytokines and growth factors, such as TGF-β, IDO, PGE2, HGF, heme oxygenase 1, IL-10 and nitric oxide, have been shown to effect on properties and biological functions of T and B lymphocytes, accompanying by inhibition of the proliferation of B cells and the activation and proliferation of T cells [41, 44–46].

It has been reported that EVs derived from MSCs can modulate the membranous expression of CD154 of intrahepatic CD4^+^ T cells, which initiates inflammatory response in liver to protect against liver ischemia/reperfusion injury (IRI) [47]. In mouse model of ALI, Liu et al. [48] demonstrated that MSCs can suppress the systemic immune response by reducing the numbers of Ly6C^low^CD8^+^T-RM cells, conventional NK cells, and IgM^+^IgD^+^ B cells. Additionally, MSCs suppressed the activation of Ly6C^hi^CD8^+^ T-RM cells and downregulated the expression of MHCII and IgM in IgM^+^IgD^+^ B cells. They also increased the number of immunosuppressive MoMF during the injured phase. Of note, MSCs promoted the retention of Ly6C^low^CD8^+^ T-RM cells and maintained the immunosuppressive activity of MoMF during the recovery phase. These findings highlight the importance of dynamically assessing of the immunomodulatory effects of MSCs. MSCs can also alleviate experimental immune-mediated liver injury, accompanying by suppressive effects on infiltration and activation of hepatic T cells, as demonstrated in animal models of Concanavalin A-induced liver injury. In this study, chitinase 3-like protein 1 secreted by MSCs inhibited the STAT1/3 signaling in T cells through upregulating peroxisome proliferator-activated receptor (PPAR) δ [49].

The crosstalk between MSCs and the hepatic inflammatory microenvironment is bidirectional. In fact, the immunoregulatory functions of MSCs vary according to the inflammatory microenvironment. For example, Deng et al. [50] demonstrated that co-infusion of human amniotic MSCs and Tregs prevented mild liver fibrosis comparing with human amniotic MSCs or Tregs alone group. Mechanically, Tregs expressed TGF-β, which activated the IDO signal of human amniotic MSCs and improved HGF secreting of human amniotic MSCs. Therefore, the biological properties and applications of MSC, especially in the liver inflammatory microenvironment, should be investigated by taking account of cell–cell interactions within the liver immune microenvironment. Although many studies have demonstrated that MSCs mainly exert immunosuppressive effects in hepatic inflammatory microenvironment, it should be noted that the immunomodulatory function of MSCs is multifaceted and highly plastic. Zong et al. [51] reported that administration of MSCs at the early stage of hepatocarcinogenesis can inhibit the development of liver cancer. However, a recent study has identified that a unique subset of inflammation-associated MSCs, known as AIF1^+^CSF1R^+^MSCs, which is present in the inflammatory microenvironment prior to the onset of liver cancer. Moreover, it is likely that this subgroup is induced by TNF-α stimulation through the TNFR1/silent information regulator 1 (SIRT1) pathway. Additionally, in a rat primary liver cancer model, MSCs with high expression of SIRT1 (Ad-Sirt1-MSCs) promoted macrophage recruitment and synergistically facilitated liver cancer occurrence by secreting C–C chemokine ligand 5 (CCL5). Interestingly, the depletion of macrophages or knockdown of CCL5 expression in Ad-Sirt1-MSCs attenuated the promotive effect of Ad-Sirt1-MSCs on liver inflammation and hepatocarcinogenesis [52]. Noticeability, the immunomodulatory ability of MSCs is not innate but is licensed and dictated by the inflammation types and intensity [53]. In fact, the inflammatory status should be taken into account before MSCs administration. In an environment characterized by low-grade inflammation, MSCs have been demonstrated to enhance the activation of T cells, consequently exacerbating the inflammatory response. Conversely, in an environment with high-grade inflammation, MSCs are shown to elicit a regulatory role in the induction of Treg cell proliferation, thereby contributing to the resolution of inflammation [53]. Therefore, the ultimate immunomodulatory impact may be determined by the balance between anti-inflammatory and pro-inflammatory cytokines in the milieu in which MSCs present.

Inflammation also plays an important role in the progression of liver fibrosis. Inflammatory cytokines released by injured hepatocytes and immune cells induce the trans-differentiation of hepatic stellate cells (HSCs) from a quiescent to a proliferative, migratory, and fibrotic phenotype [54]. Activated HSCs are the central driver of hepatic fibrosis, given their potential to induce connective tissue formation and extracellular matrix (ECM) protein accumulation. MSCs exert immunomodulatory effects in the context of liver fibrosis by modulating both innate and adaptive immune responses. It has been reported that human bone marrow-derived MSCs (BM-MSCs) transplantation rescued fulminant hepatic failure mice, as demonstrated by robust proliferation and trans-differentiation of functional hepatocytes and multiple immune cell lineages, including B cells, T cells, NK cells, DCs and macrophages [55]. Zheng et al. [56] reported that Follistatin-like 1 (FSTL1) is essential for the immunosuppressive action of MSCs on inflammatory macrophages in liver fibrotic therapy. Knockdown of FSTL1 in MSCs significantly attenuated this property through inhibiting the downstream Janus kinase/STAT1/IDO pathway. As previous discussed, in the early stages of the onset of inflammation, immune responses may be enhanced by MSCs through the release of chemokines to recruit immune cells to the hepatic environment. When exposed to adequate levels of pro-inflammatory cytokines, continuous inflammatory stimuli induce MSCs to alleviate inflammation and promote hepatic homeostasis. The dual functions of MSCs in immunomodulation may be partially responsible for the chronic inflammation during the progression of hepatic fibrosis.

### Paracrine effects

Although early interest in MSCs therapy mainly revolves around their ability to differentiate in the liver, accumulating studies demonstrated that the predominant regenerative paradigm of MSCs transplantation was the paracrine effect but not the differentiation effect. As described above, some of the therapeutic effects of MSCs mainly mediated by the secreted soluble factors. There are many soluble bioactive factors, including cytokines, chemokines, immunomodulatory molecules, and growth factors, in MSCs-CM that exert immunomodulatory functions, inhibit cell death and fibrosis, stimulate vascularization, promote tissue remodeling, and recruit other cells in various tissues. More importantly, several studies provide significant evidence that the therapeutic benefits of MSCs in chronic and acute liver disorders are systemic and that these impacts are dependent on the secretion of substances that are trophic and immunoregulatory. For example, it has been reported that MSCs-secreted PGE2 can protect against ALF via enhancing hepatocyte proliferation [57]. Moreover, Yes-associated protein (YAP) played a vital role in PGE2-triggered hepatocyte proliferation. Human UC-MSCs-CM probably improved CCl_4_-induced ALI due to its antioxidant and anti-inflammatory effects [58]. In addition, functional secretome analysis of MSCs-CM revealed Annexin-A1 as an important paracrine factor involved in immunomodulation and liver regeneration in a mouse model of ALF [59]. Additional paracrine factors, such as TSG-6, IDO, and HGF, were identified in MSCs supernatants and found to have therapeutic effects on various liver diseases that rely on their capabilities in establishment of immunosuppressive microenvironments and promoting liver regeneration.

In addition to cell–cell contact, MSCs may indirectly modulate the activity of HSCs through paracrine effects [60, 61]. HGF and nerve growth factor, which are secreted by MSCs, were shown to impair the activation of HSCs by inhibiting nuclear factor kappa-B signaling [61, 62]. TSG-6, a cytokine released from MSCs, showed significant downregulation of HSCs activation markers and upregulation of senescence markers. Organoids derived from TSG-6-treated HSCs mediated by YAP-1 can restore fibrotic liver, suggesting that direct reprogramming of HSCs by TSG-6 can be a useful strategy to control liver disease [63]. Similarly, TSG-6 interacts with CD44 is involved in fate-change of HSCs, highlighting the connection for TSG6 with CD44 to activate β-catenin and YAP-1 during the conversion of TSG-6-treated HSCs into stem-like cells [64]. Milk fat globule-EGF factor 8 have been found to be an anti-fibrotic protein in the secretome from MSCs that remarkably suppressed TGF-β signaling and reduced ECM deposition and liver fibrosis in mice [65]. Tonsil-derived MSCs-CMs can exert anti-inflammatory and anti-fibrotic effects through the endogenous production of IL-1Ra in the CCl_4_-induced liver injury model [66].

In addition, another group reported that the secretome released from miR-122-transfected adipose-derived stem cells (ADSCs) can reduce collagen content and suppress production and release of proinflammatory cytokines in the mouse model of thioacetamide (TAA)-induced liver fibrosis [67]. On the one hand, MSCs can be induced to generate a specialized secretome customized to a specific disease. On the other hand, appropriate stimulation of MSCs with pathogenic agents can lead to the production of a secretome specialized for protecting against the pathogen. For example, peroxiredoxin-1, a robust antioxidant protein, was found to be one of representative components of TAA-induced MSCs-secretome and played an important role in the protection of TAA-induced liver injury [68]. Similarly, another study demonstrated that the secretome obtained by stimulating ADSCs with disease-causing agents represented higher liver regeneration, anti-inflammatory and anti-apoptotic properties, particularly in the mouse model of hepatitis B compared to the naïve secretome [69]. Taken together, cytokines, chemokines, growth factors, ECM components, and metabolic products have shown to be functional molecules of MSCs in various therapeutic paradigms. However, there is still uncertainly in the literature regarding the biological properties and function of MSCs-derived secretory factors. Indeed, a better understanding of the mediators secreted by MSCs will not only enhance the understanding of the mechanism of action of MSCs-based therapy, but also eventually lead to the development of an optimized cocktail of crucial compounds responsible for the therapeutic effects of MSCs.

## MSCs-derived exosomes for treating liver diseases

Previous studies have shown that the EVs generated by MSCs, such as exosomes and microvesicles, could make a significant contribution to the therapeutic potential of MSCs. Exosomes are EVs with a size range of 40–160 nm in diameter with an endosomal origin, and ectosomes are generally in the size range of 5–1000 nm in diameter [70]. Exosomes are generated from the endocytic pathway, wherein budding of late endosomes leads to formation of intraluminal vesicles within multivesicular bodies that contain bioactive cargo, including protein, RNA, DNA, lipids, and metabolites [70].

Several studies revealed that EVs generated by MSCs can contribute to the therapeutic potential of MSCs by facilitating intercellular communication and delivering paracrine factors during immunomodulation, anti-fibrosis, and liver regeneration. It was reported that human UC-MSCs-derived exosome considerably suppressed hepatocyte apoptosis and reduced oxidative stress in mouse models of liver failure [71]. In vitro, it has been reported that treatment with MSCs-exosomes attenuated hepatocyte apoptosis by promoting autophagy, which was accompanied by upregulation of the expression of autophagy marker protein LC3 and Beclin-1, resulting in the promotion of autophagosomes formation [72]. Lin et al. demonstrated that MSCs exert their hepatoprotective function in a pro-autophagic dependent manner, as evidenced by increased nuclei translocation of transcription factor EB (TFEB), which promotes liver autophagy [73]. Mechanistically, let-7a-5p enriched in MSCs-exosomes can activate autophagy by targeting Mitogen-Activated Protein Kinase Kinase Kinase Kinase 3 (MAP4K3) to reduce TFEB phosphorylation. Knockdown of MAP4K3 partially mitigates the effect of anti-let-7a-5p oligonucleotide via decreasing the inflammatory response.

Between 2010 and 2019, non-alcoholic steatohepatitis (NASH) was the fastest growing cause of liver cancer deaths globally, driven by rapidly rising obesity rates and diabetes, highlighting the need for urgent measures to tackle the problem [74]. NASH arises from the confluence of multiple factors, such as genetic variants, abnormal lipid metabolism, oxidative stress, altered immune response, and imbalances in the gut microbiota [75]. Exosomes serve as mediators of intercellular communication, which are related with a wide range of pathophysiologic activities. In fact, MSCs-exosomes have emerged as vital entities in regulating tissue and intercellular metabolic signaling in during progression of NASH. Human UC-MSCs exosomes alleviate methionine/choline deficient diet-induced NASH in mice by regulating the anti-inflammatory phenotype of macrophages and by reversing PPARα protein expression in liver cells, revealing that human UC-MSCs-derived exosomes hold great potential in human MSCs-related NASH therapy. In experimental NASH animal models, human UC-MSCs exosomes could also enhance NAD(P)H quinone oxidoreductase 1 expression by promoting phosphorylation of nuclear factor erythroid 2-related factor 2, thereby improving NASH-associated hepatic inflammatory response, lipid metabolism and oxidative stress [76, 77]. MiR-627-5p encapsulated by exosomes secreted from MSCs improved glucose and lipid metabolism and alleviated liver damage, as accompanied by decreased expression of obesity-associated genes, thereby ameliorating the progression of non-alcoholic fatty liver disease [78].

Currently, mitochondrial transfer has gained considerable attention due to its critical roles in tissue homeostasis and development [79]. It has been observed in various biological contexts, such as tissue repair, cellular stress response, and intercellular communication. Notably, MSCs are frequently used as donor cells in mitochondrial transfer, indicating the potential significance of mitochondrial donation in MSCs-based cell therapy. In fact, the transfer of mitochondria between MSCs and hepatocytes has been reported. Bi et al. [80] demonstrated that the delivery of mitochondria from MSCs into steatotic cells exhibited significantly enhanced oxidative phosphorylation activity, ATP production, mitochondrial membrane potential, and reduced ROS levels in hepatocytes, which could not be achieved by the blocking of mitochondrial transfer. Similarly, the same findings were reported in NASH models, highlighting that the potential of MSCs to improve lipid load and tissue perturbance through the donation of mitochondria to the hepatocytes [81, 82].

In addition, several studies have demonstrated that human UC-MSCs-exosomes could improve hepatic glucose and lipid metabolism in rats with type 2 diabetes mellitus (T2DM) by activating autophagy [83, 84]. Li et al. [85] also discovered that human UC-MSCs can regulated lipid metabolism in T2DM mice by increasing the expression of fatty acid oxidation-related genes and inhibiting the expression of lipogenesis-related genes, which were associated with the upregulation of the hepatocyte nuclear factor 4α/carboxylesterase 2 pathway. In the same model, apoptotic vesicles derived from MSCs induced macrophage reprogramming at the transcription level in an efferocytosis-dependent manner, leading to inhibition of macrophage accumulation and transformation of macrophages toward an anti-inflammatory phenotype in the liver [86]. Overall, MSCs-derived exosomes could protect the liver by regulating glucolipid metabolism homeostasis and mitigating oxidative stress-induced liver damage.

Several studies have focused on the beneficial therapeutic effects of MSCs-derived exosomes in animal models of liver fibrosis [87, 88]. For example, the administration of MSCs-exosomes into a mouse model with schistosome-infected liver injury significantly ameliorated liver injury and enhanced the survival of these mice by decreasing the expression of α-smooth muscle actin (α-SMA), collagen I, and collagen III [89]. In the mouse model of CCl_4_-induced liver injury, MSCs-exosomes attenuated hepatic inflammation and collagen deposition by upregulating epithelial-to-mesenchymal transition-associated markers expression and downregulating collagen type I and III, TGF-β1 and phosphorylation of Smad2 [90]. Lin et al. [91] demonstrated in the same model that MSCs-exosomes treatment exerted a protective role against ferroptosis by preserving SLC7A11 function. This protective effect was accompanied by an increase in CD44 and OTU domain-containing ubiquitin aldehyde-binding protein 1 expression. Macrophages play an important role in regulating the hepatic inflammatory microenvironment. In the liver fibrosis model, MSCs-exosomes delivered miR-148a to target Kruppel-like factor 6/STAT3 pathway, leading to the differentiation of macrophages from M1 to M2 phenotype [30]. Moreover, MSCs-derived EVs are important for MSC-macrophage communications. The proteome analysis of MSCs-derived EVs revealed anti-inflammatory macrophage inducible proteins, including annexin-A1, lactotransferrin, and aminopeptidase N, following pre-conditioning with IFN-γ. This pre-conditioning altered EVs resulted in the accumulation of CX_3_CR1^+^ macrophage in the injured area and contributed to the alleviation of inflammation and fibrosis in a mouse model of cirrhosis [92]. Accumulating evidence also show that EVs derived from MSCs have therapeutic effects on liver fibrosis by inhibiting activation of HSCs. EVs from tonsil-derived MSCs ameliorated liver injury and fibrogenesis in a model of liver fibrosis, potentially by targeting hedgehog signaling pathway and attenuating HSCs activation [93]. Another study showed that MSCs-derived exosomes effectively targeted HSCs and reduced fibrosis, which was accompanied by decreased pro-fibrotic markers, such as α-SMA, collagen I. Additionally, low levels of serum circDIDO1 in these exosomes were found to correlated with liver failure clinically, and serum exosomal circDIDO1 had a well diagnostic value for liver fibrosis in patients with liver failure [94].

Liver IRI often presents in various surgical procedures such as cancer surgery or liver transplantation. Liver damage is mostly caused during reperfusion when host cells are in metabolic anaerobic distress and excess infiltration of innate immune cells occurs. Neutrophils, an essential component of IRI, are among the first cell types to be recruited from the bloodstream to injury sites, which plays an important role in fueling tissue damage by promoting the metastatic cascade through the formation of NETs [95]. Liu et al. [96] demonstrated that human UC-MSCs-EVs can protect against IRI-induced liver injury through reducing the infiltration of neutrophils and alleviating oxidative stress in rat. Another study reported that MSC-EVs exhibited a nanotherapeutic effect by inhibiting the local of formation of NETs through the transfer of functional mitochondria to intrahepatic neutrophils and the restoration of their mitochondrial function [97]. These findings underscore the therapeutic potential of UC-MSCs-EVs for liver IRI.

Multiple studies have demonstrated the potential involvement of MSCs-exosomes in liver diseases via non-coding RNAs, such as miRNA, lncRNA. Using microarray and mass spectrometry data from liver samples of patients with acute-on-chronic liver failure (ACLF), one recent study has identified that the exosomal miR-20a-5p/intrahepatocyte CXCL8 axis potentially contributes to the reduction of liver inflammation in ACLF in the context of MSCs-based therapies, highlighting that CXCL8 acts as a promising target for alleviating liver injury [98]. Another study showed MSCs-exosomes treatment could inhibit the malignant behaviors of liver cancer stem cells through the lncRNA C5orf66-AS1/microRNA-127-3p/DUSP1/ERK axis [99]. Although researchers identified evidence supporting the therapeutic effects of MSCs-exosomes, further investigation is required to elucidate the underlying mechanisms and corresponding molecular pathways. These animal model-based studies suggested that exosome secreted from MSCs may represent effective cell-free therapeutic agents for treating diverse liver diseases. Nevertheless, there have been no clinical trials conducted thus far on the use of MSCs-exosomes in the context of liver diseases. MSCs-exosome-based therapy of liver diseases still faces the challenges of rapid clearance rate and relatively short half-life in the body. In addition, the isolation of MSCs-exosomes, improvement of purification technologies and establishment of quality standards for their application remain currently unresolved. Consequently, there is a need for the development of more effective techniques for the extraction, characterization, purification, and preservation of exosomes that are applicable in therapeutic settings.

## Strategies to potentiate the therapeutic properties of MSCs in liver diseases

**Fig. 3:**
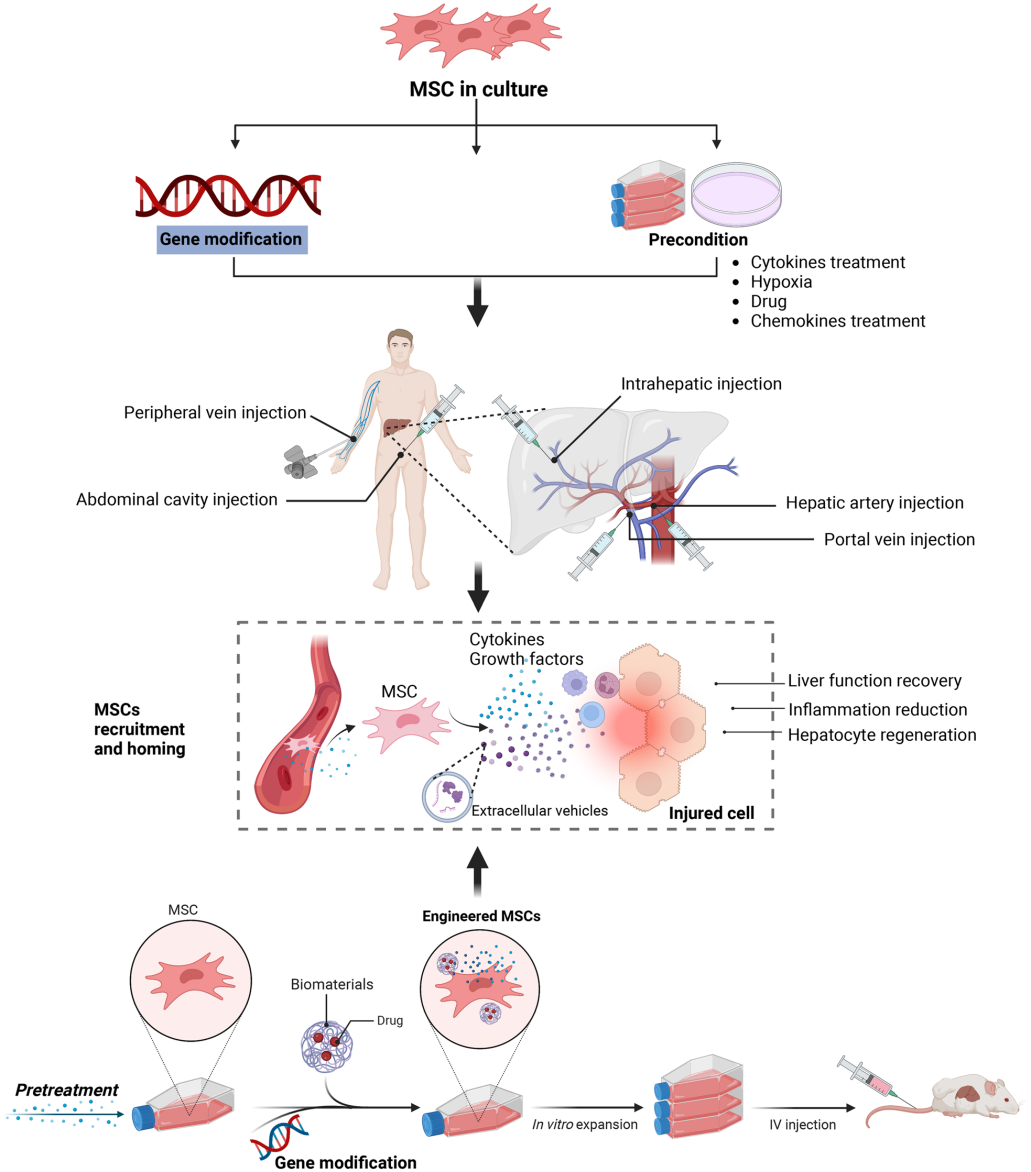
Strategies to potentiate the therapeutic properties of MSCs. Several strategies have been proposed to optimize MSCs, which can be roughly divided into two categories: pre-treatment and gene modification. Pretreated or modifying MSCs using biomaterials or bioactive substances effectively facilitates their targeted delivery to sites of liver injury. MSCs, transplanted various routes, are recruited and reach the sites of liver injury, exerting hepatoprotective, regenerative, and anti-inflammatory effects

Although several pre-clinical and clinical studies have indicated that MSCs exhibit therapeutic effects in various liver diseases, other strategies to enhance their efficacy have also been widely explored. In fact, stem cell function is regulated by both cell autonomous mechanisms as well as the niche. Stem cell niche is a complex, multidimensional system composed of both cellular and acellular components to manipulate stem cell proliferation, determine stem cell fate, and maintain stem cell homeostasis. Infused MSCs disappear soon in the recipients, which are further revealed to undergo extensive apoptosis due to the harsh microenvironment created by oxygen and nutrient deprivation and inflammation at the injured sites. Thus, the efficacy of MSCs therapy is currently limited by low retention and poor survival of transplanted cells as demonstrated by clinical studies. Considering the aforementioned immunomodulatory properties of MSCs, scientists and clinicians have made many efforts in order to enhance and control the effects of MSCs for future use in various disease treatments, which can be roughly divided into two categories: pre-treatment and gene modification (Fig. 3).

### Pretreatments

Since MSCs enter the body, they will face the pathological microenvironment created by the disease. The plasticity and memory properties of MSCs under different pathological conditions will be the primary considerations for manipulation of MSCs ex vivo. In addition, new approaches have been constantly explored for MSCs priming, including drugs and small molecule compounds. Pre-treatment with inflammatory factors and cytokines is commonly used to mimic the inflammatory microenvironment caused by the disease, which plays a significant role in regulating the immunomodulatory function of MSCs. In contrast to other factors, the production of inflammatory factors is frequently observed in the traumatic microenvironment, which can actively communicate with the resident MSCs in the tissue microenvironment. Thus, inflammatory factors have been extensively studied for pre-treating MSCs [100].

TNF-α and IFN-γ are the most common proinflammatory cytokines used for pre-conditioning MSCs, either alone or in combination. It was recently shown that the PI3K–AKT signaling axis was rapidly activated and required for skewing toward glycolysis induced by TNF-α and IFN-γ, which was critical for the production of anti-inflammation factors such as IDO1 and TSG-6 [101]. Other cytokines in the inflammatory microenvironment can also influence the directions of MSCs-mediated immunomodulation. For example, IL-17, when present together with TNF-α and IFN-γ, even at low dose, can dramatically enhance the therapeutic effects of MSCs on autoimmune hepatitis [102]. IL-1β pretreatment could partially enhance the homing ability of MSCs by increasing the expression of CXCR4, thus further improving the efficacy of MSCs on ALF [103]. Additionally, Toll-like receptor (TLR)4- and IFN-γ-activated MSCs could enhance the Th1-polarized response, which alleviated liver granulomatous and fibrosis in mice infected with *Schistosoma japonicum*, whereas TLR2- and IFN-γ-activated MSC not only induced Th1 response, but also triggered excessive inflammation, which displayed aggravated immunopathology of schistosomiasis [104]. Toll-like receptor 3 (TLR3) pre-activated BM-MSCs can have protective effects on intestinal barrier dysfunction and liver injury caused by alcohol, which may be related to its regulation of intestinal hypoxia-inducible factor (HIF) 2α gene expression and innate lymphoid cells-NKB cells [105]. Taken together, these results illustrate that MSCs can be activated by inflammatory signaling, which is sufficient to strengthen their immunoregulatory profile and therapeutic efficacy. Nevertheless, there are still unanswered questions that require intensive studies to further explore and identify the optimal concentration and duration of action for pre-treating inflammatory cytokine, either alone or in combination. Additionally, attention should be given to the possible side effects of inflammatory pre-treatment, such as undesirable upregulation of class I and II HLA molecules, which are involved in immunogenicity of MSCs.

Stem/progenitor cells are usually cultured at atmospheric oxygen tension (21% O_2_). However, physiological oxygen tension varies greatly between different tissues and can range from 3 to 12%. MSCs may experience a variety of oxygen tensions, for instance 2–7% in bone marrow or 10–15% in adipose tissue. This raises the concern that culturing MSCs at 21% O_2_ may lead to early senescence, oxidative stress, DNA damage and lower proliferation. Permanent MSCs maintenance under hypoxia should reflect the physiological conditions more adequately. Hypoxic pre-conditioning greatly modifies MSCs physiology, increasing self-renewal and survival while limiting apoptosis and senescence. Actually, hypoxia is a crucial component of the pathological setting. Existing studies also confirmed that hypoxia preconditioning and HIF-1 overexpression significantly improved MSCs therapy and MSCs cultured under hypoxic conditions presented an enhanced therapeutic effect on liver cirrhosis [106]. Thus, by mimicking hypoxia in damaged tissues, hypoxic pre-conditioning MSCs-derived secretome could improve its immunomodulatory effects. However, different tissues have different oxygen levels, different hypoxic pre-conditioning MSCs should be designed according to the specific application in order to better simulate the niche in vivo conditions required for MSCs.

Currently, two-dimensional (2D) platform is still the most commonly used for the research of cell-based assays. However, various drawbacks and limitations are still of concern. The key limitation of traditional 2D culture on tissue culture plastic is far removed from the physiological environment of MSCs. The application of the 3D culture system allows MSCs to interact with neighboring cells and the surrounding ECM, thereby influencing cellular behavior, gene expression, and paracrine signaling. MSCs spheroid have shown enhanced therapeutic potential compared to their 2D counterparts. 3D spheroid culture systems optimize the biological properties of MSCs and enable them achieve superior survival and higher differentiation efficiency. Takahashi et al. employed a human tissue to generate MSCs spheroids and observed that intrahepatic transplantation of spheroids-derived MSCs improved liver dysfunction and exhibited anti-fibrotic effects in a CCl_4_-induced liver injury model [107]. It was observed that an optimized 3D MSCs culture in spheroid forms significantly improved their paracrine effects. A study reported that elevated levels of anti-inflammatory cytokines, such as IL-10 and TGF-β1, which provide a delivery platform for the prolonged release of bioactive molecules, promoting wound healing [108]. The ECM is a 3D structure comprised of varying proteins, including collagens, elastin, and smaller quantities of structural proteins. ECM provides both structural support and dynamic microenvironment for cells regulating their behavior and fate. As a critical component of stem cell niche ECM maintains stem cells and activates their proliferation and differentiation under specific stimuli. MSCs can induce the generation of organoid structures from pluripotent stem cells through the MSCs-based traction force that is triggered by ECM proteins. MSCs can be co-cultured with primary hepatocytes on a liver-derived ECM to generate liver organoids with a short duration, which can be used to develop models for liver diseases and drug screening [109]. Additionally, hydrogels encapsulating MSCs-derived EVs can extend the bioavailability of the MSCs-EVs in the target liver, leading to improved hepatic regeneration in a model of chronic liver failure [110]. Meanwhile, as simulated ECM, hydrogels could be used as a tool to collect the secretome of MSCs merited further research.

Chemical agents with simple, cheap and efficient features also represent common pre-treatments for MSCs-based therapies for liver diseases. Rapamycin is used clinically as an antifungal treatment and for immunoregulation. The therapeutic effects and in vivo migration of rapamycin‐preconditioned UC‐MSCs have been studied. Using mouse model of liver IRI, it was noticed that, pre-conditioning of UC-MSCs by rapamycin afforded increased protection against liver IRI by enhancing immunosuppression and strengthening the homing and migratory capacity of these cells via the CXCR4/CXCL12 axis [111]. Again, preconditioning with anaesthetics, such as dexmedetomidine and midazolam, could enhance the efficacy of MSCs by increasing migratory capacity, cytokine secretion, and NF‐κB p65 nuclear translocation to protect liver cells from hypoxia‐reoxygenation-induced injury [112]. Heat shock pretreatment (HSP) is an effective way to protect cells before and after transplantation. HSP does enhance the survival rate and reduce the apoptotic rate of transplanted MSCs. Consequently, the transplantation of MSCs exposed to HSP into the portal veins of rats that had undergone hepatic IRI resulted in lower serum aminotransferase levels, lower Suzuki scores, improved histopathology and hepatocyte proliferation [113]. Melatonin is an endogenous indoleamine produced and released into the blood circulation by the pineal gland. Melatonin is a neurohormone that is primarily known as the mediator for circadian rhythms, it also presents immunomodulatory, antioxidant, and anti-aging properties [114]. Preconditioning MSCs with antioxidant melatonin enhanced their resistance to oxidative stress and led to the activation of endoplasmic reticulum (ER) stress-associated proteins and autophagy-associated proteins [115]. The ADSCs pretreated with melatonin can enhance the hepatic engraftment efficiency of these cells in liver fibrosis model by inhibiting oxidative injury [116]. Despite studies tried to improve the therapeutic effects of MSCs via multiple pathways, further studies should expand the chemical entities used to regulate MSCs for the treatment of various liver diseases.

In recent decades, a variety of bioactive compounds have been discovered and they offer a promising approach for pre-activating MSCs and enhancing their biological properties. Currently, bioactive compounds used for stimulating MSCs can categorized into natural and synthetic compounds based on their origin. The development of high-throughput technologies provides a new perspective for screening bioactive compounds that target specific genes in MSCs, thus regulating the expression profile of MSCs and specifically enhancing their desired biological functions. Given their biological mechanisms for MSCs, bioactive compounds can classify as follows: promotion of hepatogenic differentiation, enhancement of homing capability, survival and paracrine impacts. Cell model revealed that ADSCs preincubated with green tea theanine (T-ADSCs) resulted in increased cell capabilities, including viability, migration and paracrine secretion. Furthermore, T-ADSCs showed better therapeutic effect on rats with liver injury than ADSCs due to significant suppression of pyroptosis as well as autophagy accompanied with intensive paracrine vascular endothelial growth factor from T-ADSCs [117]. In addition, glycyrrhizic acid and 18β-glycyrrhetinic acid possessed diverse therapeutic properties including hepatoprotective and anti-fibrotic characteristics, which facilitated the regenerative and differentiation potential of MSCs towards hepatocytes [118]. These results indicate that they could be developed as cytoprotective agents for successful MSCs transplantation in various liver diseases. Traditional Chinese medicine (TCM) is commonly used in treating liver diseases worldwide, especially in China, and it has the following advantages: protecting hepatocytes, inhibiting hepatic inflammation and antifibrosis in the liver. In recent years, TCM and its extracts have been investigated for their beneficial effects on MSCs-based cell therapies. Baicalin, the main compound isolated from *Scutellaria baicalensis* exhibits anti-inflammatory and antioxidative effects. Exosomes derived from baicalin-pretreated MSCs were found to promote liver function recovery in mice with D-GalN/LPS-induced ALI compared to untreated MSCs. The effect was attributed to the inhibition of ROS production and lipid peroxide-induced ferroptosis [119].

Meanwhile, to further understand the role that human internal environment on exogenous infusion of MSCs in liver diseases, Zheng et al. explored the alterations in the functional properties of MSCs when exposed to ACLF serum. The study revealed that MSCs exhibited enhanced immunosuppressive function in a low-concentration serum environment, but transitioned to a proinflammatory response in a high-concentration serum environment, indicating that direct peripheral blood intravenous infusion of MSCs might diminish the effectiveness of transplantation [120]. Miyaji et al. demonstrated that co-culture of MSCs with bone marrow-derived humoral factors can suppress oxidative phosphorylation, upregulate TSG-6 expression and improve therapeutic effects of MSCs in rats with liver injury [121]. Yu et al. utilized a tissue culture plate coated with recombinant human Jagged 1 to generate Notch-activated MSCs and discovered that administration of JAG1-pretreated MSCs can mitigate APAP-induced hepatocellular damage, as manifested by reduced serum alanine aminotransferase levels, decreased infiltration of intrahepatic macrophages/neutrophils, lowered hepatocellular apoptosis and decreased production of proinflammatory mediators. Mechanically, JAG1-pretreated MSCs activated Notch2/Cyclooxygenase 2/PGE2 signaling, which subsequently induced activation of macrophage AMPK/SIRT1, leading to the X-box binding protein 1 deacetylation and the inhibition of NLRP3 activity [122].

### Gene modifications

MSCs, due to their desirable characteristics, such as multilineage differentiation, immunomodulatory property, and pro-regenerative capacity, have been used in the treatment of different types of diseases. However, the therapeutic application of MSCs faces limitations such as low survival rate, proliferation rate, and efficient migration to the target site which is probably due to the hostile microenvironment of damaged tissues and is one of the significant challenges that result in unsatisfactory therapeutic outcomes. For this reason, enhancement strategies are recommended to increase their efficiency and the EVs derived from them. One of these strategies is gene editing of MSCs. MSCs transfected with CCR2 exhibited significantly improved efficacy in the treatment of ALF in mice, which was indicated by a higher survival rate, the alleviation of liver injury with reduced inflammatory infiltration and hepatic apoptosis, and the promotion of liver regeneration [123]. Overexpression of erythropoietin promoted cell viability and migration of MSCs, leading to the alleviation of liver fibrosis [124]. Forkhead box A2-overexpressing ADSCs applied in a scaffold system could facilitate hepatocyte-like differentiation and attenuate acute liver damage without the homing effect [125]. Delivery of superoxide dismutase using MSCs as a gene delivery vehicle could reduce oxidative stress and systemic inflammation and subsequently improve glucose tolerance and fatty liver disease in diet-induced obese mouse models [126]. Furthermore, investigations have also explored the advantageous effects of genetically modified MSCs in a mouse model of high-fat diet-induced obesity, through the overexpression of IL-10. Repeated systemic administration of IL-10 modified MSCs resulted in a significant reduction in hepatic lipid accumulation [127]. Since CXCR4 and IL-10 exert independent and potentially synergistic effects on MSCs, a bicistronic mRNA-transfected MSCs carrying both the CXCR4 and IL-10 coding sequences could thus be appropriate both for improving MSCs homing to inflamed tissues and also for inducing a transient delivery of anti-inflammatory cytokines, suggesting that mRNA-modified MSCs may improve the clinical efficacy of cell therapies for the treatment of inflammatory diseases [128]. MSCs overexpressing hepatocyte nuclear factor-4 exerted good therapeutic effects against mouse liver cirrhosis due to an enhanced anti-inflammatory effect, which is likely a promising method for improving the effects of cell therapy [129]. Several studies have demonstrated that phosphatase of regenerating liver 1-overexpressing MSCs could promote liver regeneration via modulation of mitochondrial dynamics in a cirrhotic rat model, which may be linked to regulation of ER stress and calcium channels [130, 131]. MSCs modified with endogenous tissue transglutaminase have exhibited a dramatic increase in targeted efficiency to inflammatory endothelium compared with non-modified MSCs in both mice ear inflammation and acute/chronic liver injury models [132]. Researchers have employed a non-viral dual-functional nanocarrier fabricated by protamine sulfate stabilized Au nanoparticles for genetical engineering of MSCs and have also described a novel approach for enhanced therapeutic efficacy and long-term CT imaging tracking of transplanted MSCs [133].

In addition to MSCs genetically modified by coding genes, modification of microRNAs could also enhance the effects of MSCs. MiR-122 modification enhanced the therapeutic efficacy of ADSCs in the treatment of liver fibrosis by suppressing the activation of HSCs and alleviating collagen deposition [67, 134]. Furthermore, genetically modified MSCs with miR-122 can effectively package miR-122 into their secreted exosomes, which can mediate miR-122 communication between ADSCs and hepatocellular carcinoma (HCC) cells, thereby rendering cancer cells sensitive to chemotherapeutic agents [135]. Nonetheless, safety remains the primary obstacle for the prospective clinical therapeutic application of genetically modified MSCs. Moreover, the therapeutic potential and long-term function enhancements of genetically engineered MSCs require further elucidation.

## Clinical trials using MSCs or MSC-EVs in liver diseases

As of December 3, 2022, over 70 clinical trials used for liver diseases have been registered with the US National Institutes of Health (http://clinicaltrials.gov/), 11 of which have been completed and are listed (Table 1). These completed clinical trials have confirmed the therapeutic effects of MSCs in the treatment of liver diseases, particularly liver failure and liver cirrhosis. Unfortunately, although mounting evidence demonstrates that EVs derived from MSCs are potential replacements for cell-based therapies, no clinical trials have been conducted to explore the therapeutic efficacy of MSCs-derived EVs to date. In fact, as mentioned above, several unsolved issues related to the clinical application of these vesicles still exist.

**Table 1:** Clinical trials of MSCs therapy in liver diseases

Condition or disease	Design		Study phase		Source of MSCs		Injection route		Main outcome measures		Date (completed)		NCT number	
Liver failure, cirrhosis	Randomized, single group assignment		Phase 1 Phase 2		Autologous MSC		Portal vein		Liver function, MELD score, cirrhosis mortality		2009		NCT00420134	
Liver failure	Case–control		–		Autologous BM-MSC		Hepatic artery		ALT, ALB, TB, PT, MELD score, incidence of HCC		2010		NCT00956891	
Liver cirrhosis	Randomized Parallel assignment		Phase 1 Phase 2		Allogeneic UC-MSC		–		OS, liver function, incidence of HCC, Child–Pugh score, MELD score, SF36-QOL		2011		NCT01342250	
Liver fibrosis	N/A, single group assignment		Phase 1		Autologous BM-MSC		Portal vein		ALT, AST, ALB, fibrosis progression		2013		NCT01454336	
Wilson’s Disease	N/A, single group assignment		N/A		Allogeneic BM-MSC		Portal vein Hepatic artery		Liver biopsies		2014		NCT01378182	
Liver cirrhosis	N/A, single group assignment		N/A		Autologous AD-MSC		Inter-hepatic		Harmful events		2015		NCT01062750	
Liver cirrhosis	Randomized Parallel assignment		Phase 1 Phase 2		Autologous BM-MSC Allogeneic UC-MSC		Peripheral vein		Survival time, incidence of HCC events, ALT, TB, PT, ALB, PA, complications		2016		NCT01220492	
Alcoholic liver cirrhosis	Randomized Parallel assignment		Phase 2		Allogeneic BM-MSC		Hepatic artery		Safety, liver function, MELD score, histopathology, SF36-QOL, Child–Pugh score		2016		NCT01591200	
Alcoholic liver cirrhosis	Randomized Parallel assignment		Phase 2		Autologous BM-MSC		Hepatic artery		Histopathological evaluation, MELD score, Child–Pugh score, liver function (ALT, AST, ALP, ALB, bilirubin, GGT), visual inspection (liver volume, fibroscan)		2016		NCT01875081	
Liver failure	Non-randomized Parallel assignment		Phase 1 Phase 2		Allogeneic BM-MSC		Peripheral vein		Safety, tolerability, bilirubin, INR, transaminases, GGT, Biopsy-proven rejection rates, immune function		2019		NCT01429038	
Decompensated cirrhosis	Non-randomized Parallel assignment		Phase 4		Autologous MSC		Hepatic artery		Safety, MELD score, Child Pugh score, percentage of CD34 cells		2020		NCT04243681	

*ALT* alanine aminotransferase, *AST* aspartate aminotransferase, *TB* total bilirubin, *PT* prothrombin time, *ALB* albumin, *PA* prealbumin, *GGT* γ-glutamyltransferase, *ALP* alkaline phosphatase, *MELD* Model for End-stage Liver Disease, *INR* International Normalized Ratio, *MSC* mesenchymal stromal/stem cell, *BM* bone marrow, *UC* umbilical cord, *AD* adipose tissue, *OS* overall survival, *HCC* hepatocellular carcinoma

Significant differences were observed in terms of injected cell dosage, cell source, and injection route in the completed studies, with no significant adverse effects reported. Autologous BM-MSCs and allogeneic UC-MSCs are the primary cellular sources for MSCs-based therapy infusion in these clinical studies. In the clinical studies, injection routes for cell transplantation mainly include hepatic artery infusion, portal vein transplantation, peripheral vein transplantation, intrahepatic injection. In addition, cell injection methods such as intrasplenic injection, intraperitoneal injection, and intracavitary injection have been reported in other clinical studies. Indeed, these clinical studies have indicated that different cell administration routes are linked to varying cell injection dosages. Intrahepatic injection appears to be an ideal route of MSCs transplantation, as it effectively minimizes the entrapment of cells in circulation.

These studies have shown that MSCs transplantation can partially restore the liver function, ameliorate symptoms and improve the survival rates. Zhang et al. have reported that UC-MSCs therapy also significantly improved liver function in patients with decompensated liver cirrhosis, as indicated by the increase of serum albumin levels, decrease in total serum bilirubin levels, and decrease in the sodium model for end-stage liver disease scores during a 1-year follow-up period [136]. Consequently, during the 13 to 75-month follow-up period, the treated group exhibited a significantly higher overall survival rate compared to the control group. However, no significant difference in HCC event-free survival rate was observed between the treated and control groups during the 75-month follow-up [137]. An observational study involving in 513 patients who received MSCs infusion during a 3-year period further demonstrated that prolonging the treatment course can enhance the therapeutic effect of MSCs for end-stage liver disease, particularly in patients with cirrhosis [138].

Although these completed clinical studies have not shown significant occurrences of side effects or treatment-related complications, other research reports have highlighted the risk of thrombosis associated with cell infusion. A study conducted by Coppin et al. suggested that a combination of heparin and bivalirudin was added to prevent the thrombogenic risk induced by MSCs infusions in patients with Crigler–Najjar and urea cycle disorders [139]. Furthermore, another study indicated that the dosage and administration method of MSCs represent significant factors influencing the clinical outcome of MSCs therapeutics [140].

Taken together, the transplantation of MSCs has been evaluated in multiple clinical studies, and encouraging findings have demonstrated MSCs transplantation as a potential alternative method for treating patients with liver disorders. However, to strengthen the dependability of the clinical safety and efficacy of MSCs for liver disorders in humans, large-scale randomized and controlled clinical trials with extended follow-up periods are still required.

## Conclusions and future perspectives

In recent years, the advancement of regenerative medicine has positioned MSCs transplantation as a highly promising therapeutic approach for diverse liver diseases. Animal studies and clinical trials of pipeline drugs have yielded promising results in slowing disease progression. At present, conventional molecular drugs for liver diseases have displayed some limitations, such as adverse drug reactions, insensitivity to some medicines, and drug resistance [141, 142]. Extensive research has demonstrated the therapeutic potential of MSCs in liver diseases, employing various mechanisms such as immunomodulation, paracrine factors, hepatocyte-like differentiation and regeneration, anti-fibrosis and the utilization of MSCs-derived exosomes (Table 2). Furthermore, MSCs have found extensive applications in clinical research owing to their properties, such as broad sources, easy accessibility and isolation, the ability for rapid expansion in vitro, minimal biological potency loss after cryopreservation, low immunogenicity, and high levels of safety. Studies have indicated that pretreatment strategies involving inflammation milieu, hypoxia, pharmacologic agents and gene modification can effectively protect MSCs against damage in hostile environments. Consequently, these approaches enhance the homing ability, survival rate, paracrine effects both in vivo and in vitro, and therapeutic properties of MSCs in liver diseases. Numerous clinical trials have confirmed the therapeutic efficacy of MSCs in the treatment of liver diseases. Nonetheless, certain concerns with MSCs transplantation have to be addressed before their use in intensive clinical treatment despite the notable progress made in recent years.

**Table 2:** Representative studies using MSCs in experimental models of liver injury and their therapeutic mechanisms

Animal models	Source of MSCs	Doses of MSCs	Route of administration	Representative biological activities	Refs.
APAP-induced acute liver failure in mice	Human umbilical MSCs	1.0 × 10^6^	Intravenous (tail vein)	Attenuate hepatocyte necrosis by secreting hepatocyte growth factor and MDSC infiltration	[11]
TAA-injured rat model	Human PD-MSCs	2.0 × 10^6^	Intravenous (tail vein)	Trigger the regeneration of organ (e.g., liver and ovary) damaged by oxidative stress from TAA treatment via activating antioxidant factors	[16]
DGaIN-induced acute liver failure in rats	Rat bone marrow MSCs	5.5 × 10^5^	Intravenous (tail vein)	Induce M2 macrophage polarization by activating STAT6; increase the expression of anti-inflammatory factors to alleviate ALF	[24]
LPS/D-Gal-induced acute liver failure in mice	Mouse bone marrow MSCs	2.0 × 10^6^	Intravenous (tail vein)	Inhibit liver inflammatory response and hepatocyte death	[28]
CCl4-induced liver fibrosis in mice	Human umbilical MSCs	1.0 × 10^6^	Intravenous (tail vein)	Alleviate liver fibrosis and modulate macrophage phenotype to regulate inflammatory microenvironment in liver and repair the injury	[30]
CCl4-induced acute liver injury in mice	Mouse bone marrow MSCs	5.0 × 10^5^	Intravenous (tail vein)	Alleviate ALI and mitigate the recruitment of mononuclear phagocytes; attenuate the recruitment of neutrophils by reducing the expression of CXCL2 of MoMF	[31]
Ethanol-induced liver injury	Mouse bone marrow MSCs	5.0 × 10^6^	Intraperitoneal (i.p.)	Inhibit hepatic neutrophil and macrophage infiltration and alleviate oxidative stress	[35]
Ethanol-induced liver injury	Human umbilical MSCs	1.0 × 10^6^ repeated injection	Intravenous intraperitoneal	Improve survival and recovery of hepatic chemistries	[37]
Con A-induced liver injury in mice	Human umbilical MSCs	1.0 × 10^6^	Intravenous (tail vein)	Suppress T cell activation and production of pro-inflammatory cytokines through CHI3L1	[49]
TAA-induced fibrosis in mice	Human ASCs	N/A	Intravenous (tail vein)	Reduce collagen content in the liver; inhibit proinflammatory cytokines; reduce elevated liver enzymes	[59]
DDC-induced liver injury	Human MenSCs	5.0 × 10^5^	Intravenous (tail vein)	Repair the DDC-induced liver injury; inhibit COL1A1, α-SMA and TGF-β1 activation by upregulating liver β-catenin expression	[63]
CCl4-induced liver fibrosis in mice	Human umbilical MSCs	N/A	**–**	Induce ferroptosis of HSCs by regulating the xCT/GPX4 axis and alleviate liver fibrosis	[68]
CCl4-induced ACLF in mice	Human bone marrow MSCs	N/A	Intravenous (tail vein)	Restore the impaired autophagic flux and alleviate liver injury	[80]
MCD-induced NASH in mice	Human umbilical MSCs	N/A	Intravenous (tail vein)	Regulate the anti-inflammatory phenotype of macrophages and reverse PPAR-α protein expression in liver cells	[84]
T2DM-associated NAFLD in mice	Mouse bone marrow MSCs	1.0 × 10^7^ cells/kg body weight	Intravenous (tail vein)	Recover increasing weight, HFD-induced steatosis, liver function, and disordered glucose and lipid metabolism via rescuing dysfunction mitochondria	[87]
*S. japonicum*-induced schistosomiasis in mice	Human umbilical MSCs	N/A	Intravenous (tail vein)	Downregulate HSCs activation and reduce liver injury	[96]
IRI in rats	Human umbilical MSCs	3.0 × 10^6^; N/A	Intravenous (tail vein)	Alleviate hepatic ischemia–reperfusion injury by suppressing oxidative stress and neutrophil inflammatory response	[101]

*ALF* acute liver failure, *MDSC* myeloid-derived suppressor cells, *PD-MSCs* placenta-derived mesenchymal stem cells, *ALI* acute liver injury, *MoMF* monocyte-derived macrophages, *ASCs* adipose-derived stromal cells, *MenSCs* menstrual blood-derived stem cells, *HSCs* hepatic stellate cells, *ACLF* acute-on-chronic liver failure, *NASH* nonalcoholic steatohepatitis, *NAFLD* non-alcoholic fatty liver disease, *IRI* ischemia–reperfusion injury

Firstly, it is crucial to address the issue of targeted delivery (local delivery) to sites of liver damage and homing challenges. A study has shown that MSCs from which the nuclei have been removed by density-gradient centrifugation after genetic modification to express chemoattractant receptors and endothelial cell-binding molecules, serve as effective carriers for targeted delivery of therapeutics [143]. Several studies have demonstrated that modifying MSCs using biomaterials or bioactive substances effectively facilitates their targeted delivery to sites of liver injury [144, 145]. Furthermore, local administration of MSCs is increasingly recognized as a more appropriate approach for direct access to the diseased tissue and efficient release of therapeutic factors in various liver disease settings. Therefore, enhancing the homing of MSCs to the targeted sites is a strategy for augmenting the therapeutic benefits of administered MSCs.

MSCs have the potential to function as drug delivery vehicles at injury sites in liver diseases, contributing to their therapeutic effects. The soluble components derived from MSCs, such as EVs, cytokines, trophic factors, and chemokines, play a crucial role in mediating their significant therapeutic effects. MSCs-derived EVs can facilitate intercellular communication between MSCs and other cells. The regulatory functions of MSCs can also rely on paracrine mechanisms that involve coordinated interactions among various molecules delivered locally through the directed migration of cells to an injury site. Thus, the complexity associated with designing novel therapeutic strategies using MSCs must be taken into account, presenting opportunities for directing migration and engineering MSCs to deliver effector molecules at specific sites. In addition, with the deepening understanding of biomaterials, the integration of bioactive materials carrying targeted drugs may further enhance the application of MSCs-based therapy in liver diseases.

Besides, the lack of precise tracking tools to monitor the migration and survival status of MSCs within the animal body has posed a significant challenge to the clinical translation of stem cell therapies. In recent years, researchers have developed an activated near-infrared II fluorescent nanoparticle composed of lanthanide-based down-conversion nanoparticles and IR786s for cellular labeling and real-time tracking of MSCs viability in vivo. They have also identified two small molecules, glutathione and dexamethasone, that can improve MSCs engraftment efficiency and enhance the therapeutic potential of MSCs in a mouse model of liver fibrosis [146]. The tracking of intravital NIR-II photoacoustic imaging was also used to label chemokine-receptor genetically modified MSCs [147]. Furthermore, a study employed an aptamer called seq3, which exhibited high specificity and affinity for BM-MSCs, to design seq3-based activatable aptamer probes for live-cell tracking. The study revealed that the transplanted BM-MSCs were widely distributed throughout the bodies of normal mice, with a predominant aggregation observed in the livers. Moreover, they exhibited a tendency to migrate towards injured sites [148].

Additionally, there are still several challenges that need to be resolved, such as the ideal timing, optimal delivery channel, and adequate cell count for MSCs transplants. Theoretically, the quantity of MSCs influences the therapeutic efficiency of transplantation. It should be noted that the majority of infused MSCs become trapped in the lung microvasculature rather than the target tissues. Indeed, while the number of MSCs reaching the injured target organ is limited, they exhibit a potent and efficient even in small quantities [46]. Thus, further research is required to improve engraftment and the survival rate of MSCs in the liver, thereby enhancing the effectiveness of MSCs treatment. How to standardize MSCs products to achieve maximal therapeutic efficacy is another urgent issue in their clinical applications. In addition, due to the absence of specific markers for monitoring endogenous MSCs, research on the physiological roles of these cells is progressing at a slow pace. Future studies should address question related to the extent endogenous MSCs plasticity in hepatic microenvironment and to elucidate the role of endogenous MSCs in tissue homeostasis maintenance.

## Data Availability

Not applicable.

## Abbreviations

MSCs: Mesenchymal stromal/stem cells
EVs: Extracellular vesicles
ROS: Reactive oxygen species
NAC: *N*-Acetylcysteine
APAP: Acetaminophen
MDSCs: Myeloid-derived suppressor cells
HGF: Hepatocyte growth factor
CM: Conditioned medium
HPCs: Hepatic progenitor cells
NK: Natural killer
DCs: Dendritic cells
IDO: Indoleamine-2,3-dioxygenase
PGE2: Prostaglandin E2
ALF: Acute liver failure
LPS: Lipopolysaccharide
GalN: D-Galactosamine
NLRP3: NOD-like receptor thermal protein domain associated protein 3
STAT3: Signal transducer and activator of transcription 3
ALI: Acute liver injury
MoMF: Monocyte-derived macrophages
CXCL2: C-X-C motif chemokine ligand 2
NETs: Neutrophil extracellular traps
TSG6: Tumor necrosis factor stimulating gene-6
IFN-γ: Interferon-γ
Tregs: Regulatory T cells
TGF-β: Transforming growth factor-β
IL: Interleukin
IRI: Ischemia/reperfusion injury
PPAR: Peroxisome proliferator-activated receptor
SIRT1: Silent information regulator 1
CCL5: C-C chemokine ligand 5
HSCs: Hepatic stellate cells
ECM: Extracellular matrix
BM-MSCs: Bone marrow-derived MSCs
FSTL1: Follistatin-like 1
YAP: Yes-associated protein
ADSCs: Adipose-derived stem cells
TAA: Thioacetamide
TFEB: Transcription factor EB
MAP4K3: Mitogen-Activated Protein Kinase Kinase Kinase Kinase 3
NASH: Non-alcoholic steatohepatitis
T2DM: Type 2 diabetes mellitus
α-SMA: α-Smooth muscle actin
ACLF: Acute-on-chronic liver failure
TLR: Toll-like receptor
HIF: Hypoxia-inducible factor
2D: Two-dimensional
HSP: Heat shock pretreatment
ER: Endoplasmic reticulum
TCM: Traditional Chinese medicine
HCC: Hepatocellular carcinoma
